# Gene Expression Analysis of Peripheral Blood Cells Reveals Toll-Like Receptor Pathway Deregulation in Colorectal Cancer

**DOI:** 10.1371/journal.pone.0062870

**Published:** 2013-05-01

**Authors:** Ye Xu, Qinghua Xu, Li Yang, Fang Liu, Xun Ye, Fei Wu, Shujuan Ni, Cong Tan, Guoxiang Cai, Xia Meng, Sanjun Cai, Xiang Du

**Affiliations:** 1 Department of Oncology, Shanghai Medical College, Fudan University, Shanghai, China; 2 Department of Colorectal Surgery, Fudan University Shanghai Cancer Center, Shanghai, China; 3 Department of Pathology, Fudan University Shanghai Cancer Center, Shanghai, China; 4 Fudan University Shanghai Cancer Center – Institut Mérieux Laboratory, Fudan University Shanghai Cancer Center, Shanghai, China; 5 bioMérieux (Shanghai) Co. Ltd, Shanghai, China; 6 Institutes of Biomedical Sciences, Fudan University, Shanghai, China; 7 Institute of Pathology, Fudan University, Shanghai, China; University of Munich, Germany

## Abstract

Colorectal cancer is the leading cause of cancer-related deaths worldwide. The disease is curable when detected at an early stage. However, the compliance rate with current screening recommendations remains poor. An accurate, minimally invasive blood test that has the potential for greater patient compliance would be a welcome addition to the current methods. Recent data have shown that gene expression profile of peripheral blood cells can reflect disease states and thus have diagnostic value. In this study, genome-wide gene expression profiling of peripheral blood cells from 20 healthy controls and 20 colorectal cancer patients were performed using PAXgene™ technology and Affymetrix GeneChip® microarrays. We identified a list of 1,469 genes that were differentially expressed between the healthy controls and cancer patients. Gene annotation and functional enrichment analysis revealed that those genes are mainly related to immune functions. Particularly, a set of genes belonging to the Toll-Like Receptor pathways were up-regulated in the colorectal cancer patients. These findings provide a new understanding of blood gene expression profile in colorectal cancer. Our result may serve as the basis for further development of blood biomarkers for the diagnosis and treatment of colorectal cancer.

## Introduction

Colorectal cancer (CRC) is the third most common cancer in men and the second most common cancer in women worldwide. In 2008, more than 1,234,000 cases were newly diagnosed, and more than 608,000 people died from the disease [1]. Given its slow development from removable precancerous lesions and from the curable early stages, screening for CRC has the potential to reduce both the incidence and mortality of the disease [2]. The available screening tools include fecal occult blood test (FOBT), stool DNA test, flexible sigmoidoscopy, CT colonography and colonoscopy. Different screening strategies are in place in various countries. However, the compliance with current CRC screening recommendations remains poor. The low rate of participation in CRC screening is due to a number of factors, including low accuracy of current stool based screening methods, patient discomfort and poor acceptability for endoscopy based methods. An accurate, minimally invasive blood test that has the potential for greater patient compliance would be a welcome addition to the current methods. When an abnormality has been detected by the blood test, further tests involving colonoscopy and pathological examination would be recommended to confirm whether the detected abnormality is CRC.

We and others have previously showed the potential use of gene expression profiling of whole blood samples for cancer detection and diagnosis [3]–[9]. Prior to the clinical manifestation of CRC, which usually takes several years, the host reacts on the implantation of cancer cells via the activated immune system [10]. The activation of immune system is reflected in changes in gene expression profiles of immune competent blood cells, and these changes are detectable in peripheral blood [11], [12]. In this study, we performed gene expression profiling of peripheral blood cells using PAXgene™ technology and Affymetrix GeneChip® microarrays. A large number of genes differentially expressed between controls and CRC patients were identified. Particularly, we reported the overexpression profiles of Toll-Like Receptor (TLR) signaling pathways related genes in CRC to pave the way for further functional studies.

## Materials and Methods

### Patients and Sample Collection

This study was carried out at the Fudan University Shanghai Cancer Center (FDUSCC), Shanghai, China. The study was approved by the Ethical Committee of FDUSCC for clinical research. Written informed consent was obtained from all participants. Twenty CRC patients were recruited in the Department of Colorectal Surgery, FDUSCC. No patient received preoperative radiotherapy or chemotherapy. Patients suffering from hereditary CRC or inflammatory bowel diseases (Crohn’s disease or ulcerative colitis) were excluded from this study. Twenty healthy volunteers without any gastrointestinal symptoms (diarrhea and abdominal pain) were recruited through FDUSCC. All participants had blood collection at least seven days after the colonoscopy examination. For each collection, 2.5 ml of peripheral blood was drawn into a PAXgene™ Blood RNA tube (PreAnalytiX GmbH, Hombrechtikon, CH) and stored at −80°C.

### RNA Extraction and Microarray Experiments

Total RNA was extracted with the PAXgene™ Blood RNA System (PreAnalytiX GmbH). The quantity of total RNA was measured with a spectrophotometer at 260 nanometers, and the RNA integrity was assessed using an RNA 6000 Nano LabChip® Kit on a BioAnalyzer Agilent 2100 (Agilent Technologies, Palo Alto, CA, U.S.A.). All samples met the quality criterion: RNA Integrity Number >7.0 [13]. Fifty nanograms of total RNA were reversely transcribed and linearly amplified as single stranded cDNA using Ribo-SPIA™ technology with the WT-Ovation™ RNA Amplification System (NuGEN Technologies Inc., San Carlos, CA, U.S.A.), and the products were purified using the QIAquick™ PCR purification kit (QIAGEN GmbH, Hilden, Germany). Two micrograms of amplified and purified cDNA were subsequently fragmented with RQ1 RNase-Free DNase (Promega Corp., Fitchburg, WI, U.S.A.) and labeled with biotinylated deoxynucleoside triphosphates using Terminal Transferase (Roche Diagnostics Corp., Indianapolis, IN, U.S.A.) and the GeneChip® DNA Labeling Reagent (Affymetrix Inc., Santa Clara, CA, U.S.A.). The labeled cDNA was hybridized onto the GeneChip® HG U133 Plus 2.0 Array in a Hybridization Oven 640 (Agilent Technologies) at 60 rotations per minute at 50°C for 18 hours. After hybridization, the arrays were washed and stained according to the Affymetrix protocol EukGE-WS2v4 using a GeneChip® Fluidics Station 450 (Affymetrix). The arrays were scanned with the GeneChip® Scanner 3000 (Affymetrix). The microarray data have been deposited in the ArrayExpress public repository [14] with the accession number E-MEXP-3756.

### Statistical Analysis

Gene expression data analyses were performed using the R software and packages from the Bioconductor project [15]–[17]. The raw data were collected from CEL files and preprocessed using the Robust Multi-chip Average (RMA) algorithm for background correction, quantile normalization and median polish summarization [18], [19]. The probe-set-level data were log2-transformed. In addition, we applied a bioinformatics-based filtering approach using information in the Entrez Gene Database [20]. Probe sets without Entrez Gene ID annotation were removed. For multiple probe sets mapping to the same Entrez Gene ID, only probe sets showing the largest inter quantile range were kept, and the rest were excluded.

Significance Analysis of Microarrays (SAM) method [21] was used to identify genes differentially expressed between the Control and CRC groups. For gene expression studies involving microarrays, it has become common practice to focus on control of the false discovery rate (FDR), which estimates the expected proportion of incorrect rejections among the rejected hypotheses [22], [23]. To minimize false positives, we set the threshold of FDR at 0.01 for all the comparisons. Gene Ontology (http://www.geneontology.org) [24] and Panther pathway analysis (http://www.pantherdb.org/pathway) [25] were performed using the GeneCodis bioinformatics tool [26] and MetaCore™ software (GeneGo Inc., USA).

### Gene Expression Analysis by Quantitative Real-time PCR

For each sample, 200 ng of total RNA was reverse-transcribed into cDNA using Prime Script™ reverse transcriptase (TaKaRa, Dalian, China). Quantitative real-time PCR was performed by the LightCyclerH 480 system (Roche Diagnostics, Mannheim, Germany) in 96-well plates using SYBR Premix Ex Taq™ (TaKaRa, Dalian, China). Primer sequences of target genes were provided in Table S1. *CSNK1G2* (casein kinase 1, gamma 2) had previously been shown to be stably expressed in human whole blood [27], and thus was used as an internal control. The relative quantification of mRNA expression was calculated using the method described by Vandesompele *et al* [28]. Comparisons of gene expression profiles between two samples were assessed using the Welch’s t test. The significance tests were two-sided, and a *P* value below 0.05 was considered significant.

## Results

Our study included 20 CRC patients and 20 healthy controls. All the participants were Chinese, including 18 males and 22 females with a median age of 58 years (range, 42–69 years). The age and gender distributions were balanced between the Control and CRC groups. The tumors were staged according to the Tumor-Node-Metastasis (TNM) system. Two of the CRC patients were stage I, 7 were stage II, 6 were stage III, and 5 were stage IV. Detailed patient specifications are described in Table 1 and Table S2.

**Table 1:** Clinical characteristics of Controls and CRC Patients.

Characteristics	CRC	Control
**No.**	20	20
**Age - yr**		
mean	56.6	57.1
range	42–69	42–68
**Sex - no. (%)**		
male	9 (45.0)	9 (45.0)
female	11(55.0)	11(55.0)
**Tumor site - no. (%)**		
colon	9 (45.0)	–
rectum	11 (55.0)	
**Tumor stage - no. (%)**		
I	2 (10.0)	–
II	7 (35.0)	
III	6 (30.0)	
IV	5 (25.0)	

The recent release of the HG-U133plus2 microarray offers 54,000 probe sets for screening 38,500 human genes. Confronted with such an overwhelming amount of information, it was necessary to reduce the total number of genes analyzed to a manageable number of genes with verified biological annotation and use visualization schemes to facilitate the recognition of patterns in the data [29]. We thus performed a bioinformatics-based filtering procedure to summarize the probe sets at the gene level and exclude those probe sets with low-grade biological annotations. After filtering, the expression profiles of 9,529 unique genes in 20 CRC patients and 20 controls were retained for downstream analysis.

Differential Expressed Genes (DEGs) between the Control and CRC groups were identified with the SAM analysis (FDR = 0.01; Type = “Two class unpaired”; test statistic = “t-statistic”; number of permutations = 1,000). In total, 881 and 588 genes were found to be up- and down-regulated in the CRC patients. Functional enrichment analysis of Gene Ontology and the Panther pathway were carried out with a significance threshold of 0.05 for the adjusted *P* value. The Panther pathway analysis revealed a list of 22 canonical pathways that were significantly enriched in the DEG list. As expected, pathways associated with specific immune functions were well represented and highly significant, including the B cell activation, T cell activation, Interferon-gamma signaling pathway, and Interleukin signaling pathway. In parallel, several angiogenesis-related pathways including the PDGF, VEGF and FGF signaling pathways were also significantly overrepresented. The top ten associated molecular pathways and relevant genes are shown in Table 2. In addition to identifying the significant canonical pathways, we also checked genes associated with functional categories. The Gene Ontology analysis revealed a total of 74 Biological Process categories that were significantly overrepresented, including innate immune response, signal transduction, protein transport, apoptotic process, protein phosphorylation and viral reproduction. The top ten associated Biological Process categories are listed in Table 3.

**Table 2:** The top ten molecular pathways enriched in the DEG list.

Panther id	Molecularpathway	No. ofgenes	AdjustedP value	Genes
P00054	Toll receptorsignaling pathway	11	1.1E-06	*TLR2,TLR4,TANK,NFKBIA,MAP3K8,LY96,TBK1,TLR6,TLR8,MYD88,IRAK3*
P00036	Interleukin signaling pathway	10	2.0E-03	*ELK4,SOS2,MAPK6,MKNK1,IRS2,MAPK1,MAP3K2,RPS6KA3,IL13RA1,IL5RA*
P00010	B cell activation	8	2.1E-03	*NFATC3,SOS2,NFKBIA,GRAP,PPP3CA,MAPK1,MAP3K2,NFATC2*
P00053	T cell activation	9	2.3E-03	*PLCG1,B2M,NFATC3,SOS2,NFKBIA,PPP3CA,CD86,MAPK1,NFATC2*
P00047	PDGF signaling pathway	12	2.5E-03	*PLCG1,ELK4,SOS2,MAPK6,MKNK1,GRAP,JAK2,MAPK1,MAP3K2,RPS6KA3,PKN2,ETS1*
P00056	VEGF signaling pathway	8	3.7E-03	*PLCG1,PRKCH,SH2D2A,MAPK6,MAPK1,HIF1A,LPXN,ETS1*
P00021	FGF signalingpathway	10	4.8E-03	*PLCG1,PRKCH,SOS2,PPP2R5A,GRAP,MAPK1,MAP3K2,PPP2R1B,MAP2K4,PPP4R1*
P00005	Angiogenesis	12	5.0E-03	*PLCG1,PRKCH,SH2D2A,SOS2,MAPK6,TCF7L2,GRAP,MAPK1,MAP2K4,HIF1A,LPXN,ETS1*
P00018	EGF receptorsignaling pathway	10	5.3E-03	*TGFA,PLCG1,PRKCH,SOS2,PPP2R5A,GRAP,MAPK1,MAP3K2,MAP2K4,RHOQ*
P00035	Interferon-gamma signaling pathway	5	5.4E-03	*IFNGR2,PIAS1,JAK2,MAPK1,IFNGR1*

**Table 3:** The top ten biological processes enriched in the DEG list.

GO id	Biological process	No. of genes	Adjusted P value
GO:0045087	innate immune response	38	2.09E-12
GO:0007165	signal transduction	78	1.18E-11
GO:0015031	protein transport	39	1.18E-09
GO:0006915	apoptotic process	48	1.42E-09
GO:0002224	toll-like receptor signaling pathway	16	1.71E-08
GO:0006468	protein phosphorylation	36	1.88E-08
GO:0016032	viral reproduction	31	9.98E-08
GO:0002755	MyD88-dependent toll-like receptor signaling pathway	15	1.07E-07
GO:0034142	toll-like receptor 4 signaling pathway	15	1.12E-07
GO:0043123	positive regulation of I-kappaB kinase/NF-kappaB cascade	20	1.12E-07

**Figure 1:**
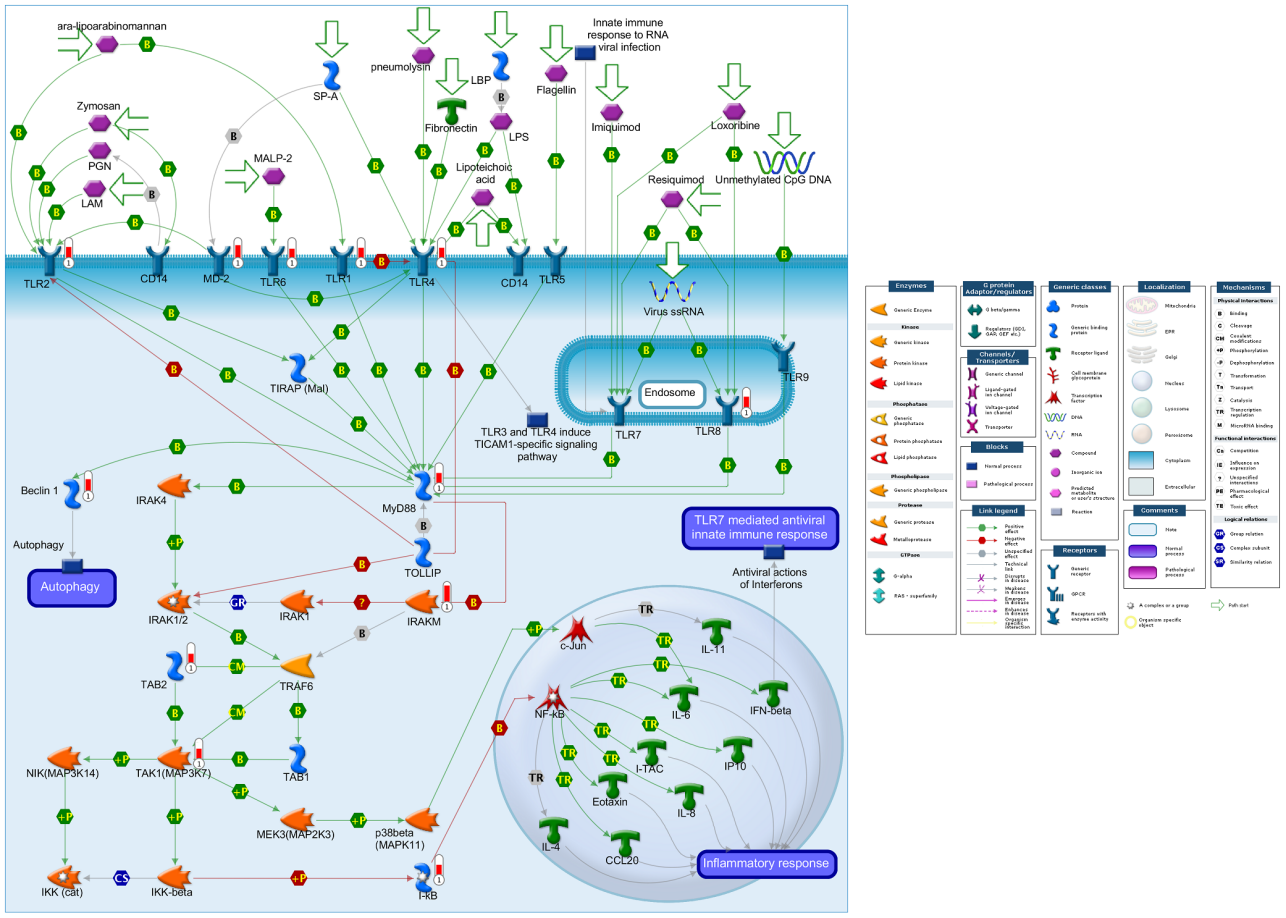
Differentially expressed genes associated with Toll-Like Receptor signaling pathways. The pathway illustration was generated and linked with available experimental data in the MetaCore™ suite. Twelve genes (*TLR1, TLR2, TLR4, TLR6, TLR8, MYD88, MD-2, Beclin 1, TAB2, TAK1, IRAKM* and *IkB)* that up-regulated in the CRC patients are linked to Toll-Like Receptor signaling pathways and can be visualized on the map as thermometer-like figures. Up-ward thermometers have a red color and indicate up-regulated levels of gene expression in the CRC patients.

Remarkably, the TLR signaling pathways were the most significantly enriched item in both the Panther pathway and GO analysis (GO0002224: toll-like receptor signaling pathway, *P = *1.7E-8; GO0002755: MyD88-dependent toll-like receptor signaling pathway, *P = *1.1E-7; GO0034142: toll-like receptor 4 signaling pathway, *P = *1.1E-7 and Panther00054: Toll receptor signaling pathway, *P = *1.1E-06). The TLR signaling pathways from the MetaCore™ software are graphically represented in Figure 1. As observed from the graph, multiple TLRs (*TLR1, TLR2, TLR4, TLR6* and *TLR8*), as well as their downstream targets, were significantly up-regulated in the CRC patients. In addition, endogenous ligands for TLRs were also identified, including *HSP70* and *HMGB1*, which have been shown to up-regulate *TLR2* and *TLR4* on tumor cell surfaces and induce tumor progression and metastasis [30], [31]. The activated TLRs then recruit *MyD88*, leading to subsequent activation of downstream targets, including *NF-κB*, mitogen-associated protein (MAP) kinase and interferon regulatory factors [32].

Quantitative real-time PCR is generally considered as the “gold-standard” assay for measuring gene expression and is often used to confirm the findings of microarray studies [33]. We thus selected six TLR signaling pathways related genes (*IRAK3, MD2*, *TLR1, TLR2, TLR4* and *TLR8*) for quantitative real-time PCR validation. The results, as shown in Table 4, indicated that gene expression profiles determined by microarray hybridization and quantitative real-time analysis were highly comparable. The gene overexpression profiles in the CRC patients were confirmed in real-time PCR data.

**Table 4:** Quantitative real-time PCR validation of TLR pathways related gene expression data.

Affy id	Symbol	Descrption	Microarray		Real-time PCR	
			Fold change	*P* value	Fold change	*P* value
210176_at	*TLR1*	toll-like receptor 1	1.43	*0.001*	1.57	*0.002*
204924_at	*TLR2*	toll-like receptor 2	1.37	*0.001*	1.32	*0.03*
232068_s_at	*TLR4*	toll-like receptor 4	1.57	*0.001*	1.37	*0.04*
229560_at	*TLR8*	toll-like receptor 8	1.50	*0.001*	1.64	*<0.001*
213817_at	*IRAK3*	interleukin-1 receptor-associated kinase 3	1.92	*<0.001*	1.68	*0.003*
206584_at	*MD2*	myeloid differentiation protein 2	1.82	*<0.001*	3.02	*<0.001*

## Discussion

Early detection of CRC is crucial for successful treatment and patient survival. However, the lack of compliance remains the greatest challenge currently limiting CRC screening effectiveness. The rich content of diverse cellular and molecular elements in blood, which provide information about the health status of an individual, make it an ideal compartment to develop noninvasive tests for CRC detection [34]. In this study, we performed global gene expression profiling of peripheral blood samples collected from 20 controls and 20 CRC patients. We identified a list of 1,469 consensus genes that differentially expressed between the controls and CRCs. Our results are consistent with previous studies [3], [5], [9] and show that most DEGs are involved in immune responses, as well as cellular apoptosis, signal transduction, protein transport and gene expression regulation.

Perhaps the most striking result to emerge from the data is the overexpression of the TLR signaling pathway related genes in the CRC patients. TLRs, the mammalian homologues of the drosophila toll protein, are the best-characterized family of pattern-recognition receptors (PRRs) [35]. To date, TLRs 1–10 have been identified in humans [36]. TLRs play a crucial role in the innate immune response and the subsequent induction of adaptive immune responses against microbial infection or tissue injury [37], [38]. Recent studies show that functional TLRs are expressed not only on immune cells but also on cancer cells, thus implicating a role of TLRs in tumor biology [39], [40]. A growing body bodies of evidence have suggested that TLRs act as a double-edged sword in cancer cells [41]. On one hand, TLRs play pivotal roles in the activation of antitumor immune responses in order to inhibit tumor progression. On the other hand, deregulated TLR signaling may provide a microenvironment that is necessary for tumor cells to proliferate and evade the immune response [42].

In particular, there have been several studies reporting an association between TLRs and colorectal neoplasia. Fukata *et al.* showed that TLR4 was overexpressed in mouse inflammation-associated colorectal neoplasia. The TLR4-deficient mice were significantly protected from colon carcinogenesis [43]. Wang *et al.* reported high expression levels of TLR4 and MyD88 associated with liver metastasis and poor prognosis in CRC patients [44]. Additionally, TLR4 and IL-6 expression in the tumor microenvironment were associated with the presence of adenocarcinoma, and higher levels of TLR4 expression in the tumor stroma were noted with disease progression [45].

As the first line of immune defense, peripheral blood cells have been shown to express all TLRs and exhibit higher levels of TLR mRNAs compared with other tissues [46]. We postulate that the up-regulation of TLR signaling-related genes in the peripheral blood is likely due to both the infiltration of TLR-expressing inflammatory cells and the up-regulation of receptor expression on these cells that occurs in response to tumor growth stimuli. Further research will be needed to understand the mechanistic relationship and the biological meanings of the overexpression of TLR pathways related genes in CRC patients. Given the therapeutic use of TLR agonists has been investigated in several cancer models [41], the systemic study of TLRs’ functions may contribute substantially to the development of new targets for the diagnosis and treatment of CRC.

In conclusion, we show that monitoring gene expression in blood results in distinct transcriptional profiles between controls and CRC patients. Thus, microarray-based blood gene expression profiling holds great promise for developing novel biomarkers for CRC detection. Future studies should include more samples for biomarker identification and validation. Furthermore, given that CRC is considered to be a genetically and epigenetically heterogeneous disease [47], it would also be interesting to investigate the blood gene expression profile of different subtypes, which may provide a new understanding of CRC.

## Supporting Information

Table S1 Primer sequences of TLR-related genes and *CSNK1G2* reference gene.
(DOCX)

Table S2 Detailed clinical information of Controls and CRC Patients.
(DOCX)
