# Correction: Gene Expression Analysis of Peripheral Blood Cells Reveals Toll-Like Receptor Pathway Deregulation in Colorectal Cancer

**DOI:** 10.1371/annotation/ed94818a-8267-4dcc-b16a-510320afb963

**Published:** 2013-10-15

**Authors:** Ye Xu, Qinghua Xu, Li Yang, Fang Liu, Xun Ye, Fei Wu, Shujuan Ni, Cong Tan, Guoxiang Cai, Xia Meng, Sanjun Cai, Xiang Du

The third author, Li Yang, should be noted as contributing equally to the work along with the first and second authors, Ye Xu and Qinghua Xu.

In the funding section, BM should not be included an an employee of bioMérieux and is not an author on this article. The correct funding statement is: "This work was supported by bioMérieux company. This study was also supported by grants from the Chinese National Clinical Key Discipline (2011–2012) and the Shanghai Science and Technology Commission of Shanghai Municipality (number 10DJ1400500). As QX, FW, FL, XY and XM are the employees of bioMérieux company and have contributed to this work, hence, as one of the funders, bioMérieux company played a role in study design, data collection and analysis, decision to publish, and preparation of the manuscript." 

