# Supplementary material for: Gene Expression Analysis of Peripheral Blood Cells Reveals Toll-Like Receptor Pathway Deregulation in Colorectal Cancer
Source: PLoS One. 2013 May 1;8(5):e62870. doi: 10.1371/journal.pone.0062870 (PMC3641099; doi:10.1371/journal.pone.0062870)
Supplement: Table S1 — Primer sequences of TLR-related genes and CSNK1G2 reference gene. (DOCX) [file pone.0062870.s001.docx]

**Table S1: Primer sequences of TLR-related genes and *CSNK1G2* reference gene.**

| ***Gene Symbol*** | ***Forward*** | ***Reverse*** |
| --- | --- | --- |
| *IRAK3* | ATGCTCGGTCATCTGTGGCAGT | CTCTGATGTTCTAGGTGGGACC |
| *MD2* | CCCTGTATAGAATTGAAAGGATCC | TGCGCTTTGGAAGATTCATGGTG |
| *TLR1* | CAGCGATGTGTTCGGTTTTCCG | GATGGGCAAAGCATGTGGACCA |
| *TLR2* | CTTCACTCAGGAGCAGCAAGCA | ACACCAGTGCTGTCCTGTGACA |
| *TLR4* | CCCTGAGGCATTTAGGCAGCTA | AGGTAGAGAGGTGGCTTAGGCT |
| *TLR8* | ACTCCAGCAGTTTCCTCGTCTC | AAAGCCAGAGGGTAGGTGGGAA |
| *CSNK1G2* | AAGGAGCGGTACCAGAAGATCG | GAAGAGCTTCCGCAGGTAGTCA |
