# Supplementary material for: Gene Expression Analysis of Peripheral Blood Cells Reveals Toll-Like Receptor Pathway Deregulation in Colorectal Cancer
Source: PLoS One. 2013 May 1;8(5):e62870. doi: 10.1371/journal.pone.0062870 (PMC3641099; doi:10.1371/journal.pone.0062870)
Supplement: Table S2 — Detailed clinical information of Controls and CRC Patients. (DOCX) [file pone.0062870.s002.docx]

**Table S2: Clinical information of Controls and CRC Patients.**

| Sample ID | Age | Gender | Tumor Site | UICC Stage | TNM classification |
| --- | --- | --- | --- | --- | --- |
| CRC_1 | 62 | F | rectal | I | T2N0M0 |
| CRC_2 | 43 | F | rectal | I | T2N0M0 |
| CRC_3 | 62 | F | colon | II | T3N0M0 |
| CRC_4 | 69 | M | colon | II | T3N0M0 |
| CRC_5 | 62 | M | colon | III | T4N1M0 |
| CRC_6 | 63 | M | colon | II | T4N0M0 |
| CRC_7 | 60 | F | rectal | II | T4N0M0 |
| CRC_8 | 60 | F | rectal | II | T4N0M0 |
| CRC_9 | 59 | M | rectal | II | T4N0M0 |
| CRC_10 | 63 | M | rectal | II | T3N0M0 |
| CRC_11 | 57 | F | colon | III | T4N1M0 |
| CRC_12 | 52 | F | colon | III | T4N2M0 |
| CRC_13 | 53 | F | rectal | III | T1N2M0 |
| CRC_14 | 47 | F | rectal | III | T4N2M0 |
| CRC_15 | 56 | M | rectal | III | T2N1M0 |
| CRC_16 | 42 | F | colon | IV | T4N2M1 |
| CRC_17 | 55 | F | colon | IV | T4N2M1 |
| CRC_18 | 61 | M | colon | IV | T4N2M2 |
| CRC_19 | 51 | M | rectal | IV | T4N2M1 |
| CRC_20 | 54 | M | rectal | IV | T4N1M1 |
| Control_1 | 53 | F |  |  |  |
| Control_2 | 51 | M |  |  |  |
| Control_3 | 55 | M |  |  |  |
| Control_4 | 59 | M |  |  |  |
| Control_5 | 57 | M |  |  |  |
| Control_6 | 67 | F |  |  |  |
| Control_7 | 64 | F |  |  |  |
| Control_8 | 67 | F |  |  |  |
| Control_9 | 64 | M |  |  |  |
| Control_10 | 43 | F |  |  |  |
| Control_11 | 64 | M |  |  |  |
| Control_12 | 59 | F |  |  |  |
| Control_13 | 65 | F |  |  |  |
| Control_14 | 67 | M |  |  |  |
| Control_15 | 43 | F |  |  |  |
| Control_16 | 45 | F |  |  |  |
| Control_17 | 42 | M |  |  |  |
| Control_18 | 57 | F |  |  |  |
| Control_19 | 68 | M |  |  |  |
| Control_20 | 52 | F |  |  |  |
